# Mast cell activation in lungs during SARS-CoV-2 infection associated with lung pathology and severe COVID-19

**DOI:** 10.1172/JCI149834

**Published:** 2023-10-02

**Authors:** Janessa Y.J. Tan, Danielle E. Anderson, Abhay P.S. Rathore, Aled O’Neill, Chinmay Kumar Mantri, Wilfried A.A. Saron, Cheryl Q.E. Lee, Chu Wern Cui, Adrian E.Z. Kang, Randy Foo, Shirin Kalimuddin, Jenny G. Low, Lena Ho, Paul Tambyah, Thomas W. Burke, Christopher W. Woods, Kuan Rong Chan, Jörn Karhausen, Ashley L. St. John

**Affiliations:** 1 Program in Emerging Infectious Diseases, Duke-NUS Medical School, Singapore.; 2 The Peter Doherty Institute for Infection and Immunity, University of Melbourne, Melbourne, Victoria, Australia.; 3 Victorian Infectious Diseases Reference Laboratory, Melbourne, Victoria, Australia.; 4 Department of Pathology, Duke University Medical Center, Durham, North Carolina, USA.; 5 Duke-NUS Medical School, Program in Cardiovascular and Metabolic Disorders, Singapore.; 6 Department of Infectious Diseases, Singapore General Hospital, Singapore.; 7 Infectious Diseases Translational Research Programme, Department of Medicine, Yong Loo Lin School of Medicine, National University of Singapore, Singapore.; 8 Division of Infectious Disease, University Medicine Cluster, National University Hospital, Singapore.; 9 Center for Applied Genomics and Precision Medicine, Duke University Medical Center, Durham, North Carolina, USA.; 10 Division of Infectious Diseases, Duke University Medical Center, Durham VA Medical Center, Durham, North Carolina, USA.; 11 Department of Anesthesiology, Duke University Medical Center, Durham, North Carolina, USA.; 12 Department of Microbiology and Immunology, National University of Singapore, Singapore; 13 SingHealth Duke-NUS Global Health Institute, Singapore.

**Keywords:** COVID-19, Mast cells

## Abstract

Lung inflammation is a hallmark of Coronavirus disease 2019 (COVID-19) in patients who are severely ill, and the pathophysiology of disease is thought to be immune mediated. Mast cells (MCs) are polyfunctional immune cells present in the airways, where they respond to certain viruses and allergens and often promote inflammation. We observed widespread degranulation of MCs during acute and unresolved airway inflammation in SARS-CoV-2-infected mice and nonhuman primates. Using a mouse model of MC deficiency, MC-dependent interstitial pneumonitis, hemorrhaging, and edema in the lung were observed during SARS-CoV-2 infection. In humans, transcriptional changes in patients requiring oxygen supplementation also implicated cells with a MC phenotype in severe disease. MC activation in humans was confirmed through detection of MC-specific proteases, including chymase, the levels of which were significantly correlated with disease severity and with biomarkers of vascular dysregulation. These results support the involvement of MCs in lung tissue damage during SARS-CoV-2 infection in animal models and the association of MC activation with severe COVID-19 in humans, suggesting potential strategies for intervention.

## Graphical abstract

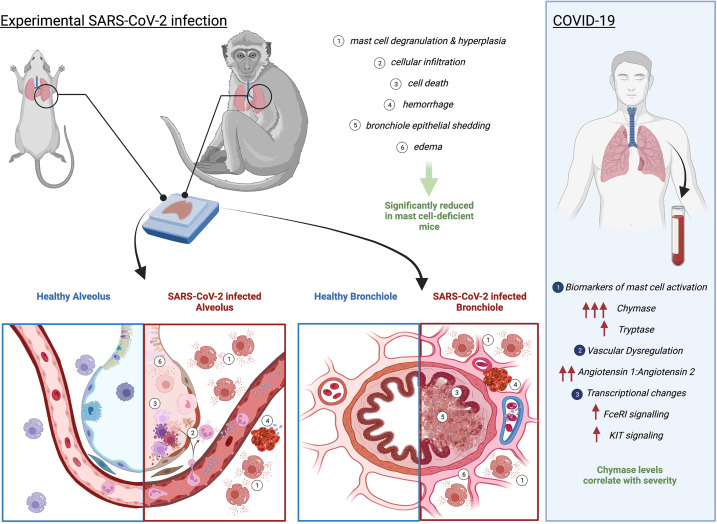

## Introduction

Coronavirus Disease 2019 (COVID-19) is caused by Severe Acute Respiratory Syndrome Coronavirus 2 (SARS-CoV-2), a recently emerged coronavirus that has resulted in an ongoing global pandemic. Clinical disease ranges from asymptomatic, to mild, to severe, and manifestations include upper respiratory tract symptoms, pneumonia, and, in some cases, acute respiratory distress syndrome (ARDS) (1). Fever, cough and anosmia are most commonly experienced at disease presentation, and complications involving the vascular system can occur during severe disease (1). The lung is a major target organ of SARS-CoV-2 infection due to abundant expression of the angiotensin converting enzyme 2 (ACE2) receptor, a cellular entry receptor for SARS-CoV-2 (2). The virus is typically shed from the nasopharyngeal tract and disseminated by coughing, but it can also be detected in fecal excretions (3). Various mouse and nonhuman primate (NHP) models have been utilized to study COVID-19 (4). NHPs and human ACE2 (*hACE2*) knock-in mice both have been shown to experience infection and recapitulate human signs of disease in the lung, including lung pathology (4). In human autopsy studies of severe disease, infiltration of mononuclear cells in the lung tissue concurrent with edema and hemorrhage are frequently described (5). It is believed that lung pathology during COVID-19 is immune mediated and compounded by the infiltration of monocytes, neutrophils, and subsets of T cells (6). Interestingly, perturbations in the numbers of granulocytes in the blood, such as neutrophils and eosinophils, have also been shown to be associated with severe disease (1, 7, 8).

Another granulocyte that responds to viral infections and is found in the lung tissue is the mast cell (MC). MCs are long-lived granulated immune cells that are present in both connective and mucosal tissues (9). In adults, MCs are thought to be derived from precursor cells circulating in the blood, known as MC progenitors, but they are only found in mature form in tissues (10), making them difficult to study in humans. Tissue-resident MCs have a mature phenotype and express a variety of pathogen recognition molecules on their surface and inside cytosolic compartments (11, 12). Their granules are loaded with preformed mediators such as histamine, serotonin, and unique MC-specific proteases, chymase and tryptase, among others. Some of their mediators, including soluble cytokines and lipid mediators, may also be produced by other granulocytes and immune cells (13). MC-derived products not only promote tissue inflammation through the recruitment of cells such as monocytes, neutrophils, and T cells, they also have substantial effects on vascular permeability and vasomotor control (9, 12, 14). The influence of MCs on vasomotor control, including vasoconstriction and vasodilation, may also contribute to hypoxia that occurs through shunting, which can influence vascular and tissue integrity (15). The tissue-specific microenvironment where a MC resides influences its phenotype. For example, MCs in the atopic lung express higher levels of the IgE receptor FcεR1 than in the skin (16, 17), and lung MCs are well characterized to contribute to pathological lung inflammation during conditions such as asthma (18). MCs are known to coordinate effective immune responses against invading pathogens, including viruses (12), but their activation has also been linked to severe tissue damage, such as during dengue virus (DENV) infection (19). In the lung, MC hyperplasia has also been reported during respiratory syncytial virus or parainfluenza virus infections (20, 21), and therapeutic stabilization of MCs was shown to reduce lung lesions in a model of highly pathogenic H5N1 influenza infection (22). However, sustained and systemic activation of MCs could also result in severe pathologies such as coagulation disorders and vascular leak. For example, MC-specific products such as chymase have been shown to be predictive of dengue hemorrhagic fever and the severity of vascular leakage and coagulopathy that characterize severe disease (19, 23, 24). MCs are present both in the nasal mucosae as well as in the deeper lung tissue where SARS-CoV-2 infection occurs; however, it is unknown whether MCs respond to highly pathogenic coronaviruses or if they could be involved in exacerbating the severe inflammation seen in SARS-CoV-2 infection.

In this study, we aimed to assess MC activation in response to SARS-CoV-2 infection. Using mouse and NHP models of COVID-19 we identified widespread MC degranulation in both acute and convalescent lung tissues. In a human cohort, prospective analysis of the transcriptional signatures of MC-precursors were highly enriched in the blood of patients who presented with severe COVID-19 disease, as were several host response pathways for prominent MC-derived products, suggesting modulation of this cell type during disease. Further, the MC-specific product chymase was significantly elevated in the sera of SARS-CoV-2 infected patients, confirming human MC activation during COVID-19 and supporting the likelihood that MCs contribute to severe COVID-19 disease.

## Results

### MC degranulation coincides with lung pathology in a NHP model of COVID-19.

**Figure 1:**
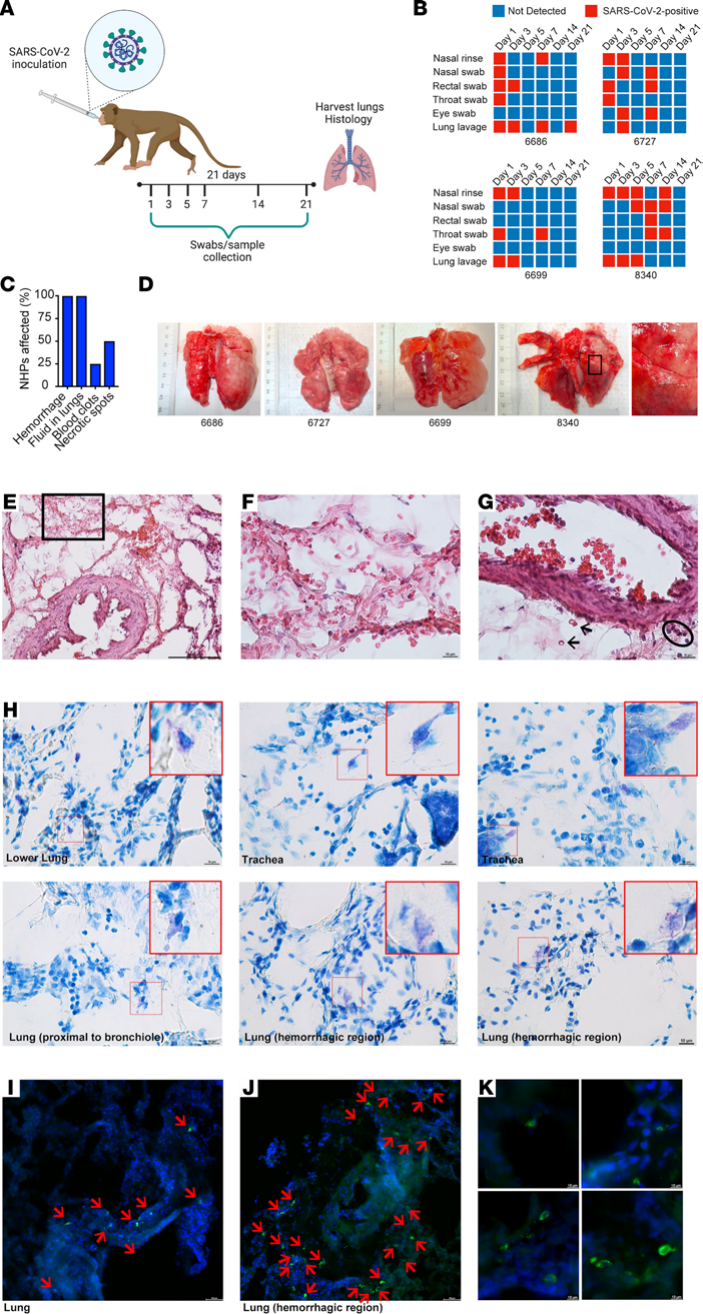
Widespread activation of MCs coinciding with lung pathology in NHPs. (**A**) Cynomolgus macaques were infected intratracheally with SARS-CoV-2 and monitored for 21 days before necropsy. (**B**) Viral detection was determined by PCR at regular intervals postinfection in swabs from multiple mucosal tissues, lung lavage, and nasal rinses. All NHPs were positive for SARS-CoV-2 infection multiple days after inoculation. (**C**) Abnormal findings related to lung tissue observed at the time of necropsy were recorded and affected all animals. (**D**) Images of NHP lungs at the time of necropsy show areas of hemorrhaging and necrotic spots on the lung surface. Boxed region is enlarged. (**E**) Histological assessment of lung tissues by H&E staining shows hemorrhaging of the tissue and free RBCs within the lung alveolar spaces. Scale bar: 100 μm.(**F**) Inset corresponding to the boxed region of **H**. Scale bar: 10 μm. (**G**) Some RBCs in the tissue proximal to a blood vessel are indicated by arrows and cellular infiltrates are circled. Scale bar: 10 μm. (**H**) Multiple examples of degranulating or hypogranulated MCs are provided, observed in toluidine blue–stained lung tissue sections. The MCs are enlarged in the red-outlined insets. Scale bars: 10 μm. For **I**–**K**, lung sections were stained for MC heparin to indicate the location of MC granules (green) and DAPI to identify cellular nuclei and tissue structures. MCs are indicated with red arrows. (**I**) MCs were observed degranulating in the lung of SARS-CoV-2 infected primates in sections of a biopsy of lung tissue that did not have overt hemorrhaging visible on the lung surface at necropsy. Scale bar: 50 μm. (**J**) MCs appear more densely packed in the lung biopsy from a hemorrhagic lobe of the lung and again, degranulation is observed based on staining for MC-heparin. Scale bar: 50 μm. (**K**) Images of degranulating MCs are presented at higher magnification. Scale bar: 10 μm.

Given the association of MCs with chronic airway inflammation, their immune sentinel role for certain viral pathogens, and the knowledge that severe lung inflammation also characterizes COVID-19, we questioned whether MCs are activated in animal models of SARS-CoV-2 infection. We first examined the MC phenotype in the NHP model, which is thought to replicate the signs and symptoms of human SARS-CoV-2 infection (4). For this, cynomolgus macaques were infected with 3 × 10^6^ TCID-50 (50% tissue culture infectious dose) of SARS-CoV-2 virus intratracheally and were monitored with minimal interventions for 21 days before necropsy (Figure 1A). Throughout the study the animals were generally active, alert, and responsive. There were no significant changes in body weight or temperature during the study (Supplemental Figure 1; supplemental material available online with this article; https://doi.org/10.1172/JCI149834DS1). Two NHPs (numbers 6699 and 6727) displayed appetite loss, and 1 was given s.c. fluids. SARS-CoV-2 could be detected in the nasal rinse or swab of all NHPs at multiple time points during acute infection, as well as in the throat swab and lung lavage at least 1 time point postinfection (Figure 1B). Additionally, 3 of 4 NHPs were positive by rectal swab and 1 also had detectable SARS-CoV-2 by eye swab (Figure 1B). In support of active infection, all NHPs seroconverted by day 14 (Supplemental Table 1). At the time of necropsy on day 21, evidence of severe lung disease was apparent, with all displaying damage to the lung tissue, including areas of hemorrhaging visible on the lungs and fluid accumulation in the lungs (Figure 1, C and D). Additionally, 1 NHP had blood clots inside the lungs, and 50% of NHPs had areas of black necrotic patches on the lungs (Figure 1, C and D), indicating severe virus-induced pathology. RNA was extracted from lung tissue from each NHP and all samples were PCR-negative for SARS-CoV-2. Interestingly, upon necropsy, NHP no. 6727 had detectible virus in the cerebrospinal fluid (CSF). These findings suggested that the NHPs in this study experienced ongoing inflammation and tissue damage even after the resolution of active infection.

Histological assessments of lung tissue showed severe damage to the airways and lung-associated vasculature that coincided with activation of MCs in tissues. Signs of hemorrhage were present in the lung tissue, where RBCs were observed in the extravascular space, both trapped within the alveoli, which were occasionally abnormally thickened (Figure 1, E and F, and Supplemental Figure 2, A–C), as well as near blood vessels (Figure 1G and Supplemental Figure 2D). Proximal to blood vessels, there was also evidence of infiltration of immune cells into the tissue (Figure 1G) and fibrin deposition (Supplemental Figure 2D). In multiple locations within the lung, including in the trachea and the lower lung lobes, as well as near bronchi and near alveolar spaces, hypodense MCs could be observed after staining of tissue sections with toluidine blue, suggesting their recent degranulation (Figure 1H). Free granules were observed extracellularly near MCs (Figure 1H), also indicating degranulation. This widespread activation of MCs was confirmed by fluorescence staining to detect heparin-containing granules in the lung tissue (Figure 1, I–K). We noted that activated MCs were especially densely located and degranulating within the hemorrhagic regions of the infected lung tissue (Figure 1, H and J). At higher magnification, free granules could be observed near hypogranulated MCs (Figure 1K), also indicating recent degranulation. These results support that SARS-CoV-2 infected NHPs experience lung pathology involving hemorrhagic manifestations and widespread MC activation, which persisted to late time points in the disease course.

### MC-dependent lung pathology during SARS-CoV-2 infection.

**Figure 2:**
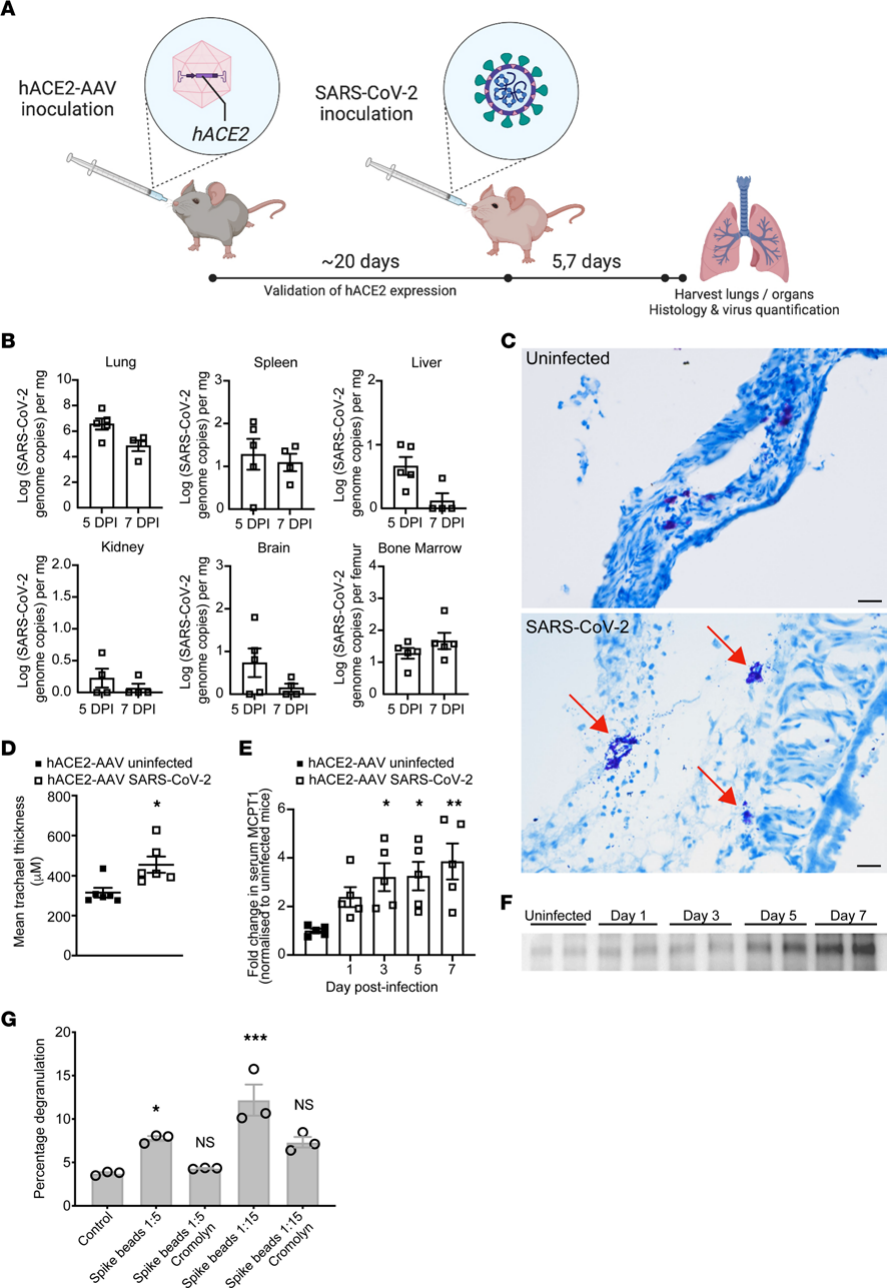
Degranulation of MCs in SARS-CoV-2 infected mice. (**A**) Experimental design of *hACE2*-AAV inoculation and SARS-CoV-2 infection in mice. C57BL/6J mice were inoculated intranasally with *hACE2*-AAV to induce *hACE2* expression in the airways. SARS-CoV-2 (2 × 10^7^ TCID_50_) was inoculated intranasally into *hACE2*-AAV C57BL/6J mice. Blood was taken on days 1, 3, 5, and 7, and organs were harvested after 5 or 7 days for histology and virus quantification. (**B**) Virus quantification from the organs harvested shows detection in the lung, spleen, liver, kidney, brain, and bone marrow from both days 5 and 7. *n* = 5 (5 days postinfection); *n* = 5 (7 days postinfection) (**C**) Representative histology images of toluidine blue–stained trachea sections from uninfected and SARS-CoV-2 infected *hACE2-AAV* mice (scale bar: 20 μm) and (**D**) mean tracheal thickness quantitated from multiple tissue sections. Degranulating MCs (red arrow) could be observed in SARS-CoV-2 infected mice as well as tissue edema and airway narrowing. (**E**) MCPT1 detection in serum Days 3, 5, and 7 postinfection shows systemic elevation of MCPT1, which was quantitated by densitometry from Western blots of 5 individual mouse samples (biological replicates) and presented as fold increase over uninfected controls. Error bars represent the SEM. MCPT1 was significantly elevated in serum of infected mice compared with uninfected controls, determined by 1-way ANOVA with Dunnett’s posthoc test where the values for each day were compared with the uninfected control; *P < 0.05, **P < 0.01. (**F**) Representative Western blot images from panel **E**. Western blots showing additional replicates are provided in Supplemental Figure 4. Expected molecular weight for MCPT1 is 28 kDa. (**G**) Dose-dependent MC degranulation in response to SARS-CoV-2 Spike protein is reduced by treatment with MC-stabilizing drug cromolyn. Purified recombinant Spike protein from SARS-CoV-2 was conjugated to 1 μm carboxylate microspheres. Significant and dose-dependent MC degranulation was induced by Spike-coated beads, but not in MCs treated with the MC-stabilizing drug cromolyn (10μM). Significance was determined by 1-way ANOVA with Tukey’s posthoc test; *P <0.05, ***P <0.001.

We next aimed to determine if MC activation promoted lung pathology during SARS-CoV-2 infection using the mouse model. To first determine whether MCs are also activated in mice during SARS-CoV-2 infection, we used an established mouse model where the receptor for SARS-CoV-2, *hACE2*, is delivered to the lungs using an adenovirus vector (AAV) (25). After *hACE2*-AAV inoculation, mice were infected with SARS-CoV-2 (Figure 2A). Blood was collected at multiple time points to assess MC-associated inflammatory products, and tissues were collected on days 5 and 7 postinfection for virus quantification by PCR. Mice showed the highest infection burden in the lungs, but for at least some of the animals, SARS-CoV-2 could also be detected in the spleen, liver, kidney, brain, and bone marrow (Figure 2B), while the brachial lymph nodes were PCR-negative at both time points (Supplemental Figure 3). Tissue histology revealed degranulation of MCs in the airways, as shown in a representative image of the trachea at day 5 postinfection (Figure 2C), where toluidine blue staining of MC granules indicated extensive degranulation that coincided with edema in the tissue. In contrast, granulated resting MCs were observed in control trachea tissue (Figure 2C). The trachea tissue from control uninfected animals also appeared healthy and compact, while the thickness of the trachea tissue in SARS-CoV-2 infected animals appears increased as a result of inflammation and swelling (Figure 2, C and D). To provide a quantitation of MC activation, we also measured serum levels of the mouse chymase MCPT1, which is a MC-specific protease that can be used as a biomarker of MC activation (19). MCPT1 levels were significantly elevated days 1, 3, 5, and 7 after SARS-CoV-2 infection (Figure 2, E and F). Some viruses or viral proteins are able to induce direct activation of MCs, while for others, MC activation can be indirect and dependent on inflammation in the surrounding tissue or antibodies (12). Since the spike protein is the major surface protein of SARS-CoV-2, we conjugated it to beads to generate particles approximately the size of virions for exposure to cultured MCs. Spike protein in this particulate form was sufficient to induce MC activation in vitro, which could be reversed by the MC-stabilizing drug cromolyn (Figure 2G). The evidence of MC degranulation in the airways combined with systemically elevated MC products indicates that SARS-CoV-2 induced substantial activation of MCs during infection in vivo and it is likely that the spike protein was responsible for direct MC degranulation, even though other host factors could also be involved in MC activation in vivo.

**Figure 3:**
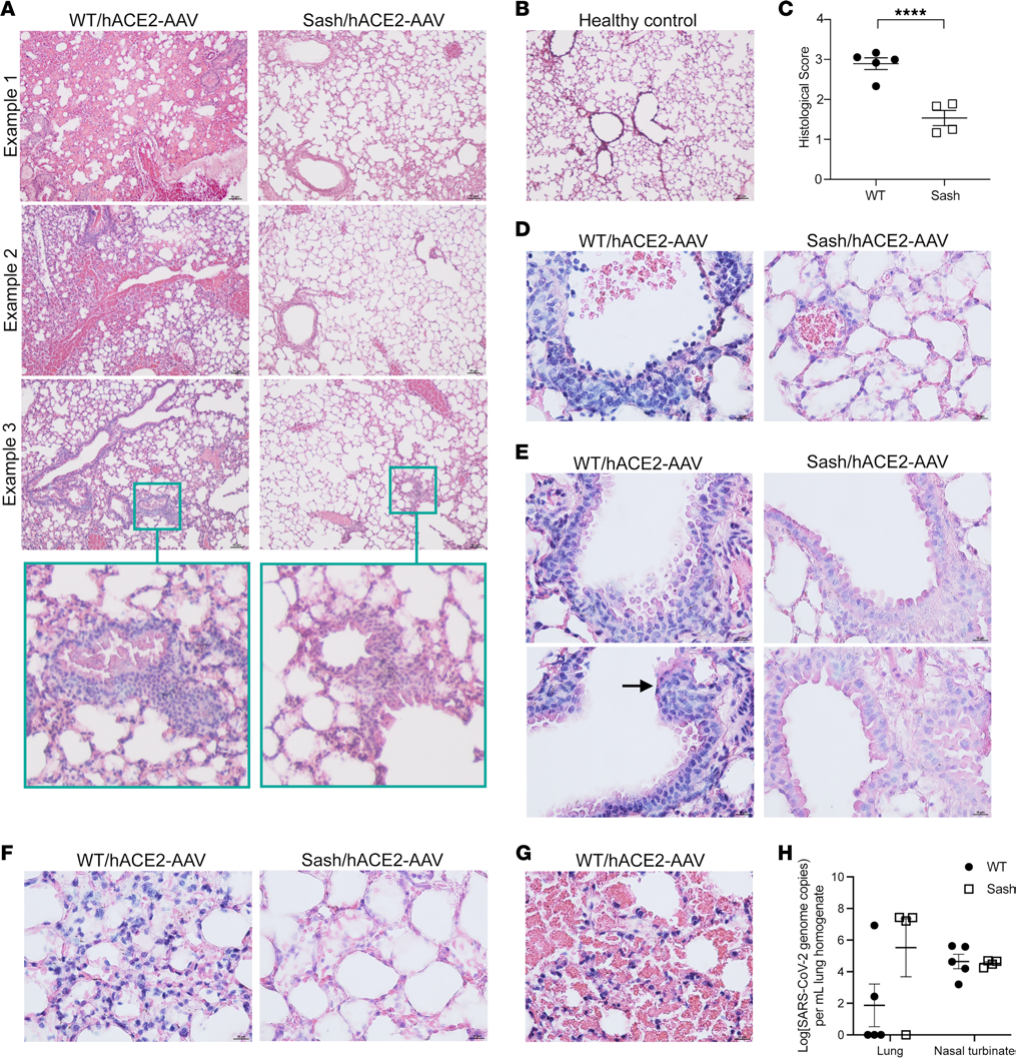
MC-dependent lung pathology in SARS-CoV-2 infection. hACE2-AAV treated WT or sash mice were inoculated intranasally with 1 × 10^5^ TCID_50_ of SARS-CoV-2/Australia/Vic/01/20 and observed daily for 5 days. *n* = 5 (C57BL/6)*; n =* 4 (*Kit^W–sh/W–sh^)*. (**A**) Three representative examples of lung tissue pathology from 3 different mice during SARS-CoV-2 infection day 5 postinfection. Insets for example 3 show areas of perivascular cuffing. (**B**) Healthy control tissue showed clear airways with no pathology. For **A** and **B**, additional representative images from infected and control groups are provided in Supplemental Figure 5A. (**C**) Histological score of SARS-CoV-2-infected mice 5 days postinfection determined by Student’s unpaired *t* test; *P* = 0.0007. Data points represent biological replicates. Examples of (**D**) venulitis with perivascular inflammation, (**E**) bronchial shedding (black arrow) and (**F**) interstitial pneumonitis in WT/hACE2-AAV infected mice, beside an image showing analogous tissue structures in sash/hACE2-AAV infected mice. (**G**) Hemorrhaging was only observed in WT/hACE2-AAV SARS-CoV-2-infected mice. (**H**) Quantification of SARS-CoV-2 genome copies in lung homogenates and nasal turbinate by PCR, which was normally distributed after log-transformation and did not differ significantly by 2-way ANOVA with Holm-Sidak’s post-test. Multiple Mann-Whitney tests on non-transformed data were also non-significant. Data points represent biological replicates. Scale bars for **A** and **B**: 50 μM; Scale bars for **D**–**G**: 10μM.

To identify whether MCs contributed directly to the pathological changes observed in the lung tissue, we compared infection in mice lacking MCs (*Kit^W–sh/W–sh^* mice, also known as “sash” mice (26–28)), to WT mice, both transfected with *hACE2*-AAV. Compared with WT/*hACE2*-AAV mice, which showed extensive edema, cellular infiltration into the tissue, hemorrhaging, perivascular cuffing, and epithelial shedding, the lung tissue from SARS-CoV-2-infected sash/*hACE2*-AAV displayed markedly reduced lung pathology (Figure 3A). Healthy lung tissue was also prepared for comparison (Figure 3B). Although reduced, inflammation characterized primarily by cellular infiltration could also be observed in some areas of infected sash/*hACE2*-AAV lungs (Figure 3A). When quantified for all animals by scoring of severity, these histological changes were significantly more severe in MC-sufficient animals (Figure 3C). At higher magnification, additional features of severity that were promoted by MCs were noted, including venulitis coinciding with perivascular inflammation (Figure 3D), bronchial cell death (Figure 3E), and interstitial pneumonitis (Figure 3F). Hemorrhaging was observed only in WT mice (Figure 3G). Although pathology in the lung tissue was significantly more severe in MC-sufficient animals, there were no statistically significant differences in viral burden in the lung tissue or nasal turbinate measured by quantitative PCR (Figure 3H) or TCID-50 (Supplemental Figure 5B). These findings lead to the conclusion that MCs are essential contributors to severe SARS-CoV-2 lung pathology in this mouse model and that features such as interstitial pneumonitis, hemorrhaging, and edema are particularly MC-dependent.

### Signatures of MC transcriptional activation are associated with severe COVID-19.

**Figure 4:**
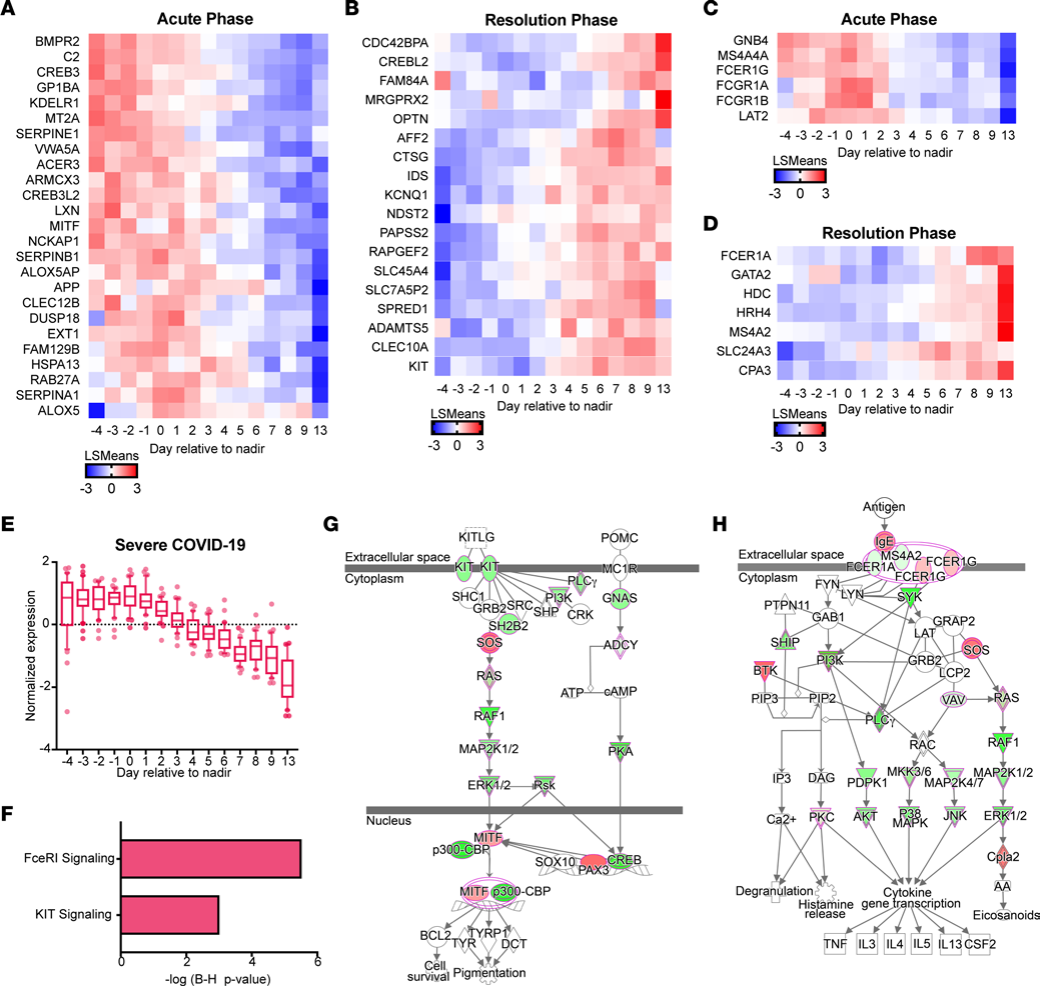
Transcriptional signatures of MC-associated genes with severe COVID-19. Genes associated with a (**A** and **B**) MC-specific or (**C** and **D**) MC/basophil phenotype that were significantly regulated in patients with severe COVID-19. Heatmap shows the LS-mean expression values of MC-specific or MC/basophil phenotype genes in patients with severe COVID-19 (n=6) at the various days relative to the peak severity with respect to respiratory function (day 0). Clusters of genes that were significantly upregulated during the acute phase (**A** and **C**) or resolution phase (**B** and **D**) are presented. (**E**) Normalized expression levels of the MC-specific genes shown in **A** and **C** over time, in the patients with severe COVID-19. (**F**) Pathway analysis indicates a significant perturbation of pathways associated with MC function and/or MC-precursor maturation. Gene network analysis for the significantly modulated pathways (**G**) KIT and (**H**) FcεRI are shown. Red indicates the genes with increased expression during the acute phase, whereas green indicates genes with increased expression during the resolution phase. *P* values for pathway and gene network analyses were generated using Ingenuity Software, which uses a right-tailed Fisher’s exact test to generate *P* values.

We then questioned whether MCs could be involved in disease in human patients with COVID-19. In healthy humans, MC precursors make up a minor component of the blood, approximately 0.005% of cells (29). MCs are known to have a unique transcriptional profile that clusters separately from other immune cells, and gene expression patterns have been identified that are either MC-specific or that typify both MCs and basophils (30). Although MCs are not present in mature form in the blood, we considered that their activation in peripheral tissues could influence the MC precursors or lead to transcriptional activation profiles in immune cells that are consistent with responses to systemically elevated MC-associated products. To investigate this, we examined whole blood transcriptomics data from a cohort of patients with COVID-19, 4 cases that were mild and 6 that were severe, where clusters of genes that were temporally modulated during severe disease progression and resolution were identified (31). Consenting patients were prospectively recruited and were defined as severe on the basis of requiring supplemental oxygen during hospitalization. In the patients who had severe disease, the gene expression levels were monitored from –4 days to 13 days, relative to the day when their condition peaked in severity of respiratory distress, which was defined as time point 0 (31). Interestingly, many genes associated with the MC lineage (Figure 4, A and B) or MC and also basophil lineages (Figure 4, C and D) were differentially modulated in the blood of human patients with COVID-19 with severe disease (*P* < 0.05; q < 0.05; likelihood ratio test). Upregulation of several genes associated with the MC- or MC/basophil transcriptional signature (30) occurred during the acute phase of severe disease (Figure 4, A and C), while others were differentially regulated at the time of disease resolution (Figure 4, B and D). The increased MC gene expression changes that were observed during the acute phase of disease tracked tightly with respiratory function and resolved commensurate with respiratory improvement (Figure 4E). In contrast, these MC-associated transcripts were not collectively changed temporally throughout the period of monitoring in mild COVID-19 presentation (Supplemental Figure 6, A–E), although some genes that were associated with these signatures were still modulated, but to a lesser extent than in severe patients (Supplemental Figure 6, A–D). Pathway analysis of the temporally modulated genes over the disease course of patients who are severely ill revealed significant perturbation of pathways downstream of key MC-associated immune receptors. (Figure 4F) One such receptor was KIT (Figure 4G), the receptor for stem cell factor, which is an important stem cell-associated gene that is retained on MC precursors and mature MCs and regulates MC survival and proliferation (32). Another receptor was FcεRI (Figure 4H), which is upregulated with MC maturation, although also expressed by other cell types such as basophils (29, 30). These data show an enrichment of MC-associated transcripts in patients with severe COVID-19 and support a potential role of MCs in shaping disease severity.

### Confirmation of MC activation in patients with COVID-19.

**Figure 5:**
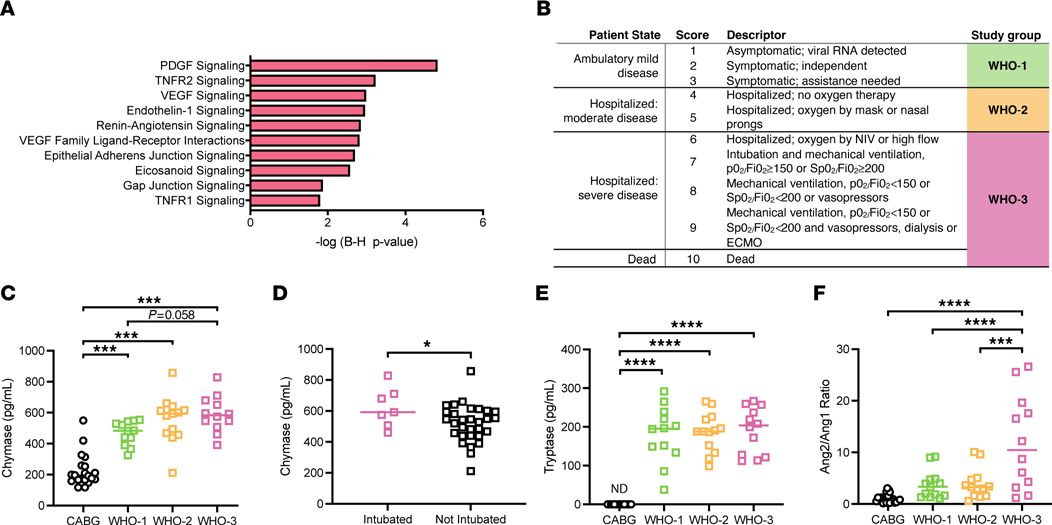
Serum chymase and MC activation pathways associated with severe COVID-19. (**A**) Pathway analysis indicates a significant perturbation of pathways associated with host responses to characteristic MC products. Ingenuity software was used to generate *P* values. (**B**) Strategy for grouping of patients by WHO score (34) for analysis. (**C**) Plasma chymase levels were increased in patients with COVID-19 compared with CABG controls and correlate with disease severity (Supplemental Figure 7A). (**D**) Chymase levels were also increased in patients who later required intubation, compared with nonintubated patients from multiple groups (WHO-1, WHO-2, and WHO-3). (**E**) Serum tryptase levels were significantly increased in all patients with COVID-19 and were undetectable in CABG controls. (**F**) Significantly increased plasma Ang2/Ang1 ratios in patients in group WHO-3 compared with other groups and CABG controls. 1-way ANOVA (**C**, **E**, and **F**) or Student’s unpaired *t* test (**D**) were performed; *P <0.05; ***P <0.001; ***P <0.0001. Nonsignificant *P* values are indicated on the graphs; significant *P* values are indicated with asterisks**.** For panels (**C**–**F**), CABG patients, *n* = 20; For WHO-1, *n* = 13; for WHO-2, *n* = 13; for WHO-3, *n* = 12.

We noted that, in addition to the significant modulation of pathways associated with MC identity and maturation (Figure 4, F–H), pathway analysis of the whole blood transcriptomics from patients with severe disease also revealed significant modulation of pathways associated with responses to well-established MC products (Figure 5A). For example, Gap and adherens junction signaling, which are influenced by MC proteases to promote vascular permeability (14), were activated, as was signaling downstream of important, albeit not cell-specific, MC products, such as VEGF, TNF, Endothelin 1, and Eicosanoids (Figure 5A). We also noted a significant influence on the renin-angiotensin pathway (Figure 5A), which is intriguing, since the MC-specific protease, chymase, mediates ACE-independent angiotensin II production (33). The modulation of these pathways regulated by MC-derived products was suggestive of MC activation, although this required direct confirmation.

To confirm the activation of MCs in humans, we measured plasma chymase levels in 2 other separate cohorts of patients with COVID-19. In the first cohort, the WHO 10-point median clinical disease severity (34) in patients was 6 (interquartile range: 5–7.25), including 3 patients with lethal outcomes. For analysis, we defined 3 groups of patients according to clinical disease severity (Figure 5B): group 1 (WHO-1) as patients having WHO scores 1–3 with ambulatory mild disease (i.e., asymptomatic to mild symptomatic disease needing assistance); group 2 (WHO-2) as patients with WHO scores 4–5 who were hospitalized with moderate disease (i.e. hospitalized needing no oxygen or only via mask or nasal prongs); and group 3 (WHO-3) as patients with scores 6–10 who were hospitalized with severe disease (including those needing oxygen by noninvasive ventilation or high flow and also including patients needing mechanical ventilation with signs of organ failure). For non-COVID-19 controls, we obtained baseline plasma samples (after the induction of anesthesia, before incision) from patients who underwent coronary artery bypass graft (CABG) surgery. This cohort was chosen since they have many of the risk factors of patients with COVID-19 and are of a similar age. In both mild and severe COVID-19 cases, the plasma sample was collected at the time of diagnosis for the majority of patients. Patient demographics are provided in Supplemental Table 2 and comorbidities are provided in Supplemental Table 3. These results indicated that patients with COVID-19 have significantly higher levels of plasma chymase compared with CABG control cases, with the highest levels detected in the WHO-3 group with severe disease (Figure 5C). Indeed, there was a significant positive correlation between disease severity according to WHO classification and plasma chymase concentration (Supplemental Figure 7A, **P = 0.0087). The WHO-3 group included 7 patients who were intubated, all but 1 of which had samples collected before intubation. Intubated patients also had significantly higher chymase levels compared with all other patients (Figure 5D). We also measured another MC-protease, tryptase, which is particularly expressed by mucosal phenotype MCs (28) but is thought to be a weaker biomarker of MC activation because of its shorter half life in vivo (35). Our results showed that tryptase was released during COVID-19 disease, but its levels did not correlate with severity in these patients (Figure 5E). Tryptase could not be detected in the samples from patients who underwent CABG (Figure 5E). We also recruited a smaller number of patients with COVID-19 in Singapore. Indeed, this second cohort of patients with COVID-19 also had elevated chymase and tryptase that were much higher than healthy controls and also averaged higher than the concentrations detected in patients with acute dengue (Supplemental Figure 7, C and D). These data were not stratified by severity due to the smaller cohort size but support the activation of MCs in human patients with COVID-19 in an independent cohort. Taken together, human chymase detection confirms that elevated chymase and heightened MC activation are associated with severe COVID-19.

Published reports highlight the importance of microvascular abnormalities in defining COVID-19 severity (36) and are supported by the demonstration of alveolar edema and hemorrhagic lesions in our murine and NHP models. MC activation has direct impact on vascular function and integrity and, therefore, we tested if MC activation was linked to vascular barrier dysfunction. For this, we measured Angiopoietin-1 (Ang1) and -2 levels as markers of endothelial activation, which are strongly linked with disease severity in ARDS (37) and COVID-19 (37). We found no change in Ang1 levels depending on WHO severity group, and lower levels than those found in CABG controls (Supplemental Figure 7D), yet higher Ang2 levels (Supplemental Figure 7E), resulting in higher Ang2/Ang1 ratios (Figure 5F) in WHO-3 group COVID-19 cases. Chymase levels were positively correlated with Ang2/Ang1 ratio (Supplemental Figure 7F, *P = 0.0319). These results suggest that there may be a link between heightened chymase levels and vascular dysregulation during COVID-19.

## Discussion

Our results indicate that MCs are strongly activated by SARS-CoV-2 infection in vivo in animal models and that their levels of activation are significantly associated with severe COVID-19 disease in humans. The activation response involves a degranulation and release of MC-associated preformed mediators, which was confirmed visually by imaging of tissue sections as well as quantitatively by detection of MC-specific chymase in the serum. MCs are present in the lung tissue, even before birth, and they are important for regulating lung tissue inflammation during homeostasis and disease (38). The heightened levels of persistent activation of MCs that we detected through the acute phase of natural and experimental SARS-CoV-2 infections are likely to be important for amplifying inflammation, which could be detrimental to recovery from infection and return to tissue homeostasis following infection clearance. In mouse and NHP lung tissue, MCs were observed to be strongly degranulating, and their increased density and morphological appearance of activation was associated with areas of tissue damage characterized by edema, hemorrhaging, and necrosis. In primates, MC hyperplasia was also observed, which is consistent with recent reports describing MC hyperplasia and activation in human lung tissues from patients with COVID-19 (39, 40). We observed that the Spike protein of SARS-CoV-2 was sufficient to induce MC activation, which has also been observed by others who further determined that the receptor binding domain was insufficient to induce this degranulation in cultured MCs (41). Together, this evidence indicates that degranulation of MCs likely occurs in lung tissue in response to the virus particle itself, although MCs may also be activated in vivo by other endogenous factors, since MC degranulation responses can occur via Fc receptor–mediated mechanisms, as well as factors such as complement split products (28).

In mice, we functionally evaluated the contributions of MCs to SARS-CoV-2–induced disease severity and determined that MC-deficiency reduced lung tissue damage. We also observed that the presence of MCs was particularly associated with interstitial pneumonitis, hemorrhaging, and edema. While MC-deficient mice also showed some evidence of inflammation and cellular recruitment to the tissue, evidence of perivascular cuffing and pneumocyte death were also reduced in the absence of MCs. The severe pathological changes observed in WT compared with sash mice occurred during SARS-CoV-2 infection even though there was not a significant difference in viral titers detected between these 2 groups. However, it should be noted that MCs often promote early clearance and containment of virus in other systems (12). We cannot exclude the possibility that there may be experimental conditions where MCs impact viral burden, even though it was not observed in this experiment. Along those lines, in a system using a pseudovirus expressing Spike protein, MCs may have influenced the distribution of the pseudovirus in the respiratory tract (42), which would suggest that they could also influence infection and antigen exposure in the lung. However, the tissue damage caused by MCs was consistent with their role in mediating inflammation and pathology in the lung that was suggested by mouse models of highly pathogenic influenza (22, 43). The role of MCs in epithelial shedding is also consistent with what has been observed with respect to the bladder epithelium (44). The persistent activated state observed in the lungs of NHPs, with sustained evidence of MC activation at the relatively late time point postinfection, when samples were no longer PCR-positive for virus detection, suggests that ongoing inflammation in the tissue may occur even after infection has resolved systemically. The late-phase activation of MCs subsequent to infection clearance and establishment of a humoral adaptive immune response could suggest a role for MCs in the sustained inflammatory response that limits disease resolution. Consistent with this, all NHPs seroconverted by 3 weeks after SARS-CoV-2 infection and beginning as early as 1 week postinfection. Interestingly, IgGs targeting various self antigens, including IFNs, phospholipids and cytokines, as well as heightened total IgE levels, have been detected in patients with severe COVID-19 (45–48). MCs respond to antibodies in unique ways when triggered by antigen/antibody immune complexes. Classically, known for its activation by crosslinking of IgE-FcεRI in the presence of an antigen, MCs can also be activated by IgG immune complexes, owing to their surface expression of activating FcγRs (49–51). In addition to genes that are consistent with a MC-specific transcriptional profile, we also found significant upregulation of pathways typifying both MCs and basophils (30) in patients with severe compared with mild COVID-19, such as multiple Fc receptors. Our observation is consistent with the association of FCER1 transcriptional regulation recently reported to be associated with severe COVID-19 by others (52). Whether MCs can also be activated by autoantibodies that are evoked in the absence of an active infection remains to be elucidated and might be relevant to long COVID-19 with persistent symptoms.

We also observed transcriptional responses of patients with severe COVID-19 that coincide with peak respiratory distress and that point to the enhanced function or abundance of cells having a MC-like phenotype. Pathways characteristic of proinflammatory responses of responder cells to MC-derived products were also modulated. Since mature MCs are not present in the blood (10) and only present in tissues, the MC-associated phenotype described here is likely more consistent with MC precursors than with mature MCs. We noted that transcripts of proteins that are specific to mature granulated MCs, such as chymase, were not identified as a component of the MC-associated transcriptional profile that was induced along the time course of peak severity. These results are highly suggestive of the expansion or increased maturation of MC precursors in the blood, but further studies are needed to fully understand the responses of this cellular compartment to infection. A limitation here is the potential to only monitor transcriptional responses in the human blood, yet our animal model data supports that there is expansion of MCs in the lung tissue as well. This is consistent with the observation in a murine model of H1N1 influenza infection where recruitment and maturation of MC progenitors in the lung was suggested to occur approximately 2 weeks after the infection (43). We also noted an unusually high density of MCs in damaged and hemorrhagic regions of NHP lungs at 3 weeks after SARS-CoV-2 infection. Increased transcriptional upregulation of the chemokine CXCR2 in the blood of patients with severe COVID-19 is also suggestive of MC precursor migration into lung, as was seen in the context of other diseases (53). Similarly, other studies have identified transcriptional signatures of granulocyte activation as well as increases in cells such as neutrophils, eosinophils and basophils, and T cells in the blood or lung tissue itself in severe COVID-19 (1, 6–8). As tissue-resident cells, MCs are considered sentinels and they can promote the trafficking of many of these cell types into tissues during both allergic and infection-induced inflammation (9, 12, 54).

Aside from the lung-associated pathologies of COVID-19, some individuals also experience other hematological changes and cardiovascular events, including intravascular coagulation, endothelial damage with ischemic complications, the development of rashes that could be accentuated by damaged microvasculature, and increased incidence of myocardial infarction (1, 55). These effects on the vasculature and cardiovascular system are also consistent with the effects of MCs in other sterile inflammatory conditions. MCs line the blood vessels within tissues (14), which not only places them in a location where they can directly exert their effects on the vasculature, but also where their mediators can gain access to the blood. We observed that lung SARS-CoV-2 inoculation in mice and humans both results in increased levels of MC-specific chymase on a systemic level. In the renin-angiotensin system, MC-chymase is a potent converter of angiotensin I to angiotensin II, which regulates microvascular blood flow and systemic blood pressure (56–58). However, production of chymase by MCs is also associated with vascular diseases. For example, in the atherosclerotic aorta, angiotensin II activity was largely ACE-independent and dependent on chymase (59), and increased expression of chymase in the lung was associated with early pulmonary vascular disease (60). Notably, higher levels of angiotensin II in the plasma of patients with COVID-19 are correlated with lung injury suggesting its involvement in the tissue damage (61). Moreover, angiotensin II could increase the expression of endothelial-specific receptor tyrosine kinase (TIE2) ligand Ang2 (62). An imbalance of Ang2/1 is known to be associated with vascular leakage and coagulation in other diseases (63). We observed increased plasma levels of Ang2 and increased ratio of Ang2/Ang1 in patients with severe COVID-19 compared with patients with milder COVID-19 or individuals in the healthy control group. Interestingly, we also observed that transcriptional responses of the angiotensin pathway were substantially perturbed in the peripheral blood of patients with severe COVID-19. As exemplified by the endothelial activation in severe COVID-19, this highlights a potential causal role of MC activation in critical features of COVID-19 disease, including abnormalities of pulmonary blood flow leading to shunting and hypoxemia or loss of endothelial integrity leading to tissue edema. Notwithstanding its role as an angiotensin-converting enzyme, a more direct effect of chymase in cleaving endothelial tight junctions or potential contributions of other MC products, such as tryptase and serotonin (12), in COVID-19–related vascular pathologies cannot be ruled out. As such, we found intriguing parallels to DENV as another virus that induces MC activation. Although DENV does not specifically infect the lung, DENV infection is also characterized by increases in microvascular permeability and bleeding, which are augmented through the actions of MCs. It is noteworthy that, in dengue, MCs play an important role in limiting virus burden in early disease but drive clinical deterioration in disseminated disease (12). As a result, drugs targeting MCs and their products are promising as a therapeutic strategy to prevent severe clinical courses in DENV infection and may bear similar promise in preventing severe COVID-19, which warrants further evaluation. While retrospective studies suggest that antihistamine use may influence COVID-19 hospitalization rates (64, 65), and a trial of Histamine-1/Histamine-2 receptor blockade in patients with COVID-19 suggested some improvements in lung function (66), histamine is only one product produced by MCs and it is also produced by many other cell types during inflammation (14). A trial involving combination therapy of the MC-stabilizing drug ketotifen with the NSAID indomethacin is also underway (NCT05007522) and this may shed light on the potential of broadly targeting MC functions to improve COVID-19 outcomes.

## Methods

Additional methods are available in Supplemental Methods.

### Statistics.

Excel and Prism software packages were used for statistical analyses, as indicated in the figure legends. Data were considered significant when *P* < 0.05. For student’s *t-*tests, unpaired, 2-tailed tests were used. One- or 2-way ANOVAs were used to determine statistical significance involving multiple groups, as indicated in the figure legends. For Figure 5, a statistical analysis to confirm appropriate power was achieved was performed using SPSS and G*power software and outputs are provided in the Supplemental Statistical Analysis subsection.

### Study approval.

All mouse and primate studies were approved by the SingHealth IACUC of the SingHealth Experimental Medicine Centre (IACUC no.: 2020/SHS/1564) or by the Animal Welfare committee at the University of Melbourne (24818). The data associated with human transcriptional responses were approved by the SingHealth Combined IRB (CIRB 2017/2374). Patients with COVID-19 were recruited at Duke University in accordance with protocols reviewed and approved by the Duke University Health System IRB (Pro00100241), while studies of human patients with COVID-19 in Singapore were approved by the Domain Specific Review Board, Domain E for National University Hospital (no. 2020/00120) and the National University of Singapore IRB (NUS-IRB-2021-186).

### Data availability.

The raw data for the microarray profiling are available at Array Express (E-MTAB-9721). Raw data for the manuscript are also available in the Supporting Data Values file or from the corresponding author upon request.

## Author contributions

JYJT, DEA, APSR, AO, CKM, WAAS, and JK performed experiments and data analyses. Data were interpreted by JYJT, DEA, APSR, KRC, JK, and ALS. ALS and APSR wrote the manuscript with contributions by JYJT. The primate study was designed, funded, and conducted by DEA with contributions from AEZK and RF. Histological assessments in mice and primates were done by APSR. *hACE2*-AAV mouse model was established and validated by CQEL, CWC, and LH. Transcriptional analysis was performed by KRC with additional interpretation by ALS. Human clinical sample collection and patient assessments were performed by JGL, SK, PT, TWB, and CWW. Funding for the study was obtained by LH, JK, and ALS. The project was conceived and supervised by ALS. All authors reviewed the manuscript and provided feedback on it.

## Supplementary Material

Supplemental data

Supporting data values

## Version 1 08/10/2023

In-Press Preview

## Version 2 10/02/2023

Electronic publication
